# Nonaqueous Interfacial Polymerization-Derived Polyphosphazene Films for Sieving or Blocking Hydrogen Gas

**DOI:** 10.1021/acsapm.2c02022

**Published:** 2023-02-09

**Authors:** Farzaneh Radmanesh, Alberto Tena, Ernst J. R. Sudhölter, Mark A. Hempenius, Nieck E. Benes

**Affiliations:** † Membrane Science and Technology Cluster, Faculty of Science and Technology, MESA^+^ Institute for Nanotechnology, University of Twente, P.O. Box 217, 7500 AE Enschede, The Netherlands; ‡ The European Membrane Institute Twente, Faculty of Science and Technology, University of Twente, P.O. Box 217, 7500 AE Enschede, The Netherlands; § Surfaces and Porous Materials (SMAP), Associated Research Unit to CSIC, UVainnova Bldg, Po de Belén 11 and Institute of Sustainable Processes (ISP), Dr. Mergelina S/n, University of Valladolid, 47071 Valladolid, Spain; ∥ Organic Materials & Interfaces, Department of Chemical Engineering, Faculty of Applied Sciences, Delft University of Technology, 2629 HZ Delft, The Netherlands; ⊥ Sustainable Polymer Chemistry, Faculty of Science and Technology, MESA^+^ Institute for Nanotechnology, University of Twente, P.O. Box 217, 7500, AE Enschede, The Netherlands

**Keywords:** hydrogen separation, hydrogen barrier, nonaqueous interfacial polymerization, oxygen barrier, polyphosphazene, thermal stability

## Abstract

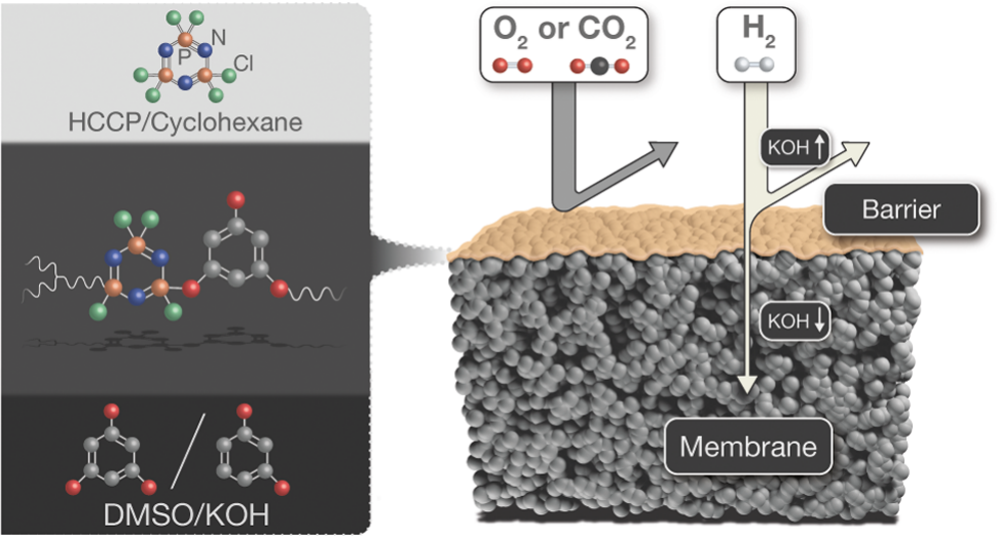

A series of cyclomatrix polyphosphazene films have been prepared by nonaqueous interfacial polymerization (IP) of small aromatic hydroxyl compounds in a potassium hydroxide dimethylsulfoxide solution and hexachlorocyclotriphosphazene in cyclohexane on top of ceramic supports. Via the amount of dissolved potassium hydroxide, the extent of deprotonation of the aromatic hydroxyl compounds can be changed, in turn affecting the molecular structure and permselective properties of the thin polymer networks ranging from hydrogen/oxygen barriers to membranes with persisting hydrogen permselectivities at high temperatures. Barrier films are obtained with a high potassium hydroxide concentration, revealing permeabilities as low as 9.4 × 10^–17^ cm^3^ cm cm^–2^ s^–1^ Pa^–1^ for hydrogen and 1.1 × 10^–16^ cm^3^ cm cm^–2^ s^–1^ Pa^–1^ for oxygen. For films obtained with a lower concentration of potassium hydroxide, single gas permeation experiments reveal a molecular sieving behavior, with a hydrogen permeance of around 10^–8^ mol m^–2^ s^–1^ Pa^–1^ and permselectivities of H_2_/N_2_ (52.8), H_2_/CH_4_ (100), and H_2_/CO_2_ (10.1) at 200 °C.

## 1 Introduction

Interfacial polymerization (IP) is a versatile and robust technique for synthesizing functional polymers in diverse forms, including nanofibers, capsules, and ultrathin polymer films.^1,2^ In conventional IP, a polycondensation reaction takes place at the interface between two immiscible solvents. Because both of the solvents contain only one of the two highly reactive monomers for the reaction, the reaction is localized to the vicinity of the interface.^3^ The film formation is affected by an intricate interplay of the reaction and diffusion of the monomers. A variety of options exist to tune the morphologies and chemistries of the films that are formed, for instance, the choice and concentrations of the monomers, the temperature, the duration of contact between the solutions, etc.

One of the key factors affecting the IP process is the selection of the solvents.^4−8^ For thin-film synthesis, and in particular for thin-film composite membranes, generally water and an organic solvent are used. By varying the organic solvent and adding cosolvents to the aqueous phase, nanofilms with the desired properties can be obtained.^4−8^ The number of studies in which water is substituted by an organic solvent is limited.^9−12^ Wamser et al. showed the formation of various polyamide porphyrin films at the interface of dimethylsulfoxide (DMSO)/chloroform and dimethylsulfoxide (DMSO)/ethyl acetate.^9^ Ogata et al. reported IP in nonaqueous systems to be useful for synthesizing aromatic polyesters and copolyesters.^10^ Heßbrügge and Vaidya demonstrated the formation of highly solvent-resistant polyamide coatings at the interface of salt crystals at 7 °C.^11^ Liu et al. presented IP at an alkane/ionic liquid interface to synthesize selective polyamide nanofilm membranes.^12^ These studies concentrate on polyamides and polyesters; to our knowledge, no attempts have been made to expand nonaqueous IP to other chemistries, except for our recent works on POSS and cyclomatrix poly(phenoxy)phosphazenes.^13,14^

These hybrid materials comprise unsaturated rings of alternating phosphor and nitrogen atoms that are covalently connected via diphenyl oxide bridges. The organic phase monomer used in the IP process is hexachlorocyclotriphosphazene (HCCP), a versatile building block for making polyphosphazene-based materials via reactions with a broad range of nucleophiles.^15,16^ The cyclic phosphazene group offers extraordinary intrinsic properties, such as natural flame retardancy and thermal stability.^17^ Most of the preparation methods involving HCCP are based on solution polymerization, and only a few studies have been dedicated to synthesizing polyphosphazene films with IP.^13−16,18^ Maaskant et al. reported that the interfacial polycondensation of HCCP with diphenols in conventional IP is too slow to allow for ultrathin (∼10^8^ m) film formation. In our previous work, we replaced the water phase with a solution of KOH in DMSO.^14^ DMSO is a polar aprotic solvent that is immiscible with organic solvents such as cyclohexane and, in addition, facilitates nucleophilic reactions between monomers. In the solution of KOH in DMSO, the diphenols are (partly) deprotonated and become very soluble and reactive nucleophilic aryloxide anions, enabling the formation of ultrathin polymer films that have a molecular sieving ability at very high temperatures.

Here, we further explore the potential of nonaqueous IP for tailoring polyphosphazene films by exchanging the diphenols for the smaller aromatic hydroxy compounds (AHCs), *p*-dihydroxybenzene (PDHB), *m*-dihydroxybenzene (MDHB), and 1,3,5-trihydroxybenzene (THB); see Table 1. These AHCs are planar molecules with the OH slightly out of the plane of the phenyl ring.^19^ AHCs are affordable and easily accessible, but they show low solubility in water. Similar to diphenols, AHCs are highly soluble in DMSO, and their hydroxyl groups can be converted into anions by adding a strong base such as KOH. The structures of AHCs allow us to synthesize a tightly packed polyphosphazene network, and the adjustable parameters of IP, such as the molar ratio of hydroxyl groups of monomer to the base, provide opportunities to tune the film morphology and cover the range of applications to selectively separate and block hydrogen as a valuable clean fuel.^20−22^

**Table 1:**
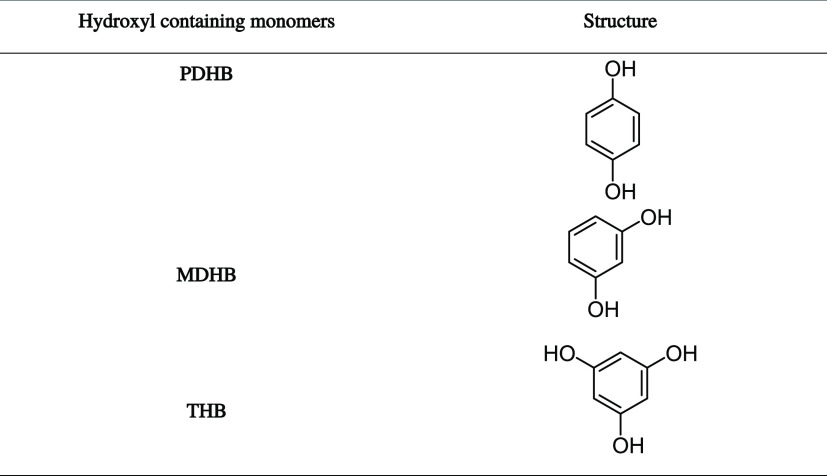
Chemical Structure of the Hydroxyl-Containing Monomers (AHCs) Used in This Study

Several studies have shown the potential of THB and PDHB to react with HCCP in a single solvent and form microspheres and microtubes.^23−25^ To the best of our knowledge, our work is the first in which AHCs and HCCP are combined to form thin-film polyphosphazene networks with properties that can be tailored from highly hydrogen-selective membranes to very tight gas barriers. The results can aid the further development of ultrathin membranes/barriers via nonaqueous interfacial polymerization with inexpensive monomers.

## 2 Experimental Section

### 2.1 Materials

Hexachlorocyclotriphosphazene (HCCP, 99%), 1,3,5-trihydroxybenzene (THB or phloroglucinol, ≥99%), *m*-dihydroxybenzene (MDHB, ≥99.9%), *p*-dihydroxybenzene (PDHB, ≥99.9%), and dimethylsulfoxide (DMSO, anhydrous, ≥99.9%) were obtained from Sigma-Aldrich. Cyclohexane (EMSURE for analysis) and potassium hydroxide (KOH, pellets extra pure) were supplied from Merck. All chemicals and solvents were used as received. Macroporous α-alumina disks with a diameter of 39 mm, a thickness of 2 mm, and a pore size of 80 nm were obtained from Pervatech B.V. and used as the support.

### 2.2 Material Fabrication

Free-standing polymeric layers and thin-film composite membranes were formed by interfacial polymerization of a 10 w/v % solution of the AHCs in super base^26^ DMSO and KOH, with a 3.5 w/v % HCCP solution in cyclohexane. The molar ratio of monomer hydroxyl groups to KOH is indicated by *x* and was kept at 4:1, 3.5:1, and 2.2:1 in DMSO unless mentioned otherwise. A higher concentration of KOH led to the formation of the phloroglucinol potassium salt and subsequently solid particles in the AHC/DMSO solution, and with a lower concentration of KOH (*x* = 6), no layer was formed at the interface of the two immiscible phases.

#### 2.2.1 Synthesis of Free-Standing Polymer Films

Free-standing films were prepared by heating the AHC solutions at 80 °C for 2.5 h, and subsequently, the HCCP solution in cyclohexane was poured on top of it. The reaction at the interface of the two solutions was confirmed by visual observation of the formation of the thin film. After 15 min, the film was collected, filtered, and washed with acetone, ethanol, and water and dried in a vacuum oven at 50 °C overnight.

#### 2.2.2 Preparation of Thin-Film Composites

A mesoporous γ-Al_2_O_3_ intermediate layer with a pore size of 5 nm and a thickness of 4–5 μm was prepared on the α-Al_2_O_3_ disks based on a previously reported procedure.^27^ The IP films were prepared on top of ceramic supports using a setup described elsewhere.^13^ First, the AHC solution and support were heated separately to 80 °C for 2.5 h and 30 min, respectively. After this, 5 mL of the AHC solution was poured on top of the support and placed in a closed box in the oven at 80 °C. After 10 min, it was taken out, and its surface was dried by applying a rubber roller and N_2_ gun. Then, 5 mL of the HCCP solution was poured atop the support at ambient temperature. After the reaction for 10 min, the solution was discarded, and the resulting thin-film composite (TFC) was rinsed with ethanol. The TFC was kept in a fume hood overnight and then dried in a vacuum oven at 50 °C for a minimum of 24 h.

### 2.3 Material Characterization

A field emission scanning electron microscope (FE-SEM, JSM-7610F) was used to visualize the thickness and morphology of the obtained TFCs. Both surface and cross-section images were acquired. Samples were prepared by immersion in liquid nitrogen for 5 min and carefully fractured to reveal the complete cross-section. Subsequently, all samples were mounted on an FE-SEM holder using a double-sided carbon tape. Fourier transform infrared spectroscopy in attenuated total reflectance mode (FTIR-ATR, PerkinElmer Spectrum Two) was used to characterize the free-standing film product. Spectra were averaged over 16 scans with a resolution of 4 cm^–1^ over a wavenumber range of 400–4000 cm^–1^. The elemental composition of synthesized free-standing films was measured with X-ray fluorescence (XRF) (S8 Tiger, Bruker) and CN elemental analysis (FLASH 2000 series analyzer). The thermal stability of the materials was measured by heating a fixed amount of sample (10 mg) on a heating stage under an inert nitrogen atmosphere at a heating rate of 10 °C min^–1^ using a thermo gravimetric analyzer (TGA, STA 449 F3 Jupiler, Netzsch) in combination with a mass spectrometer (MS, QMS 403 D Aeolos MS, Netzsch). Ultraviolet–visible (UV–vis) spectra of THB and MDHB solutions with different concentrations of KOH were recorded on a PerkinElmer λ12 UV–vis spectrophotometer.

### 2.4 Gas Permeance

Single gas permeance measurements were performed in dead-end mode using a commercially available Convergence Inspector Poseidon gas permeation setup. The single gas permeance of He (0.255 nm diameter), H_2_ (0.289 nm diameter), CO_2_ (0.330 nm diameter), N_2_ (0.364 nm diameter), and CH_4_ (0.389 nm diameter) was measured at a transmembrane pressure of 2 bar within the temperature ranges from 50 to 250 °C. The detection limit of the convergence setup was limited to 10^–10^ mol m^–2^ s^–1^ Pa^–1^. For this reason, TFC samples with low permeance were measured with a different setup,^28^ at ambient temperature and a higher transmembrane pressure of 3 bar. In this setup, the permeate side of known volume *V* [m^3^] was placed under vacuum (*p*_start_), and the gas was collected until an end pressure (*p*_end_) was achieved. The amount of collected gas *n* was calculated based on the ideal gas law, *n* = (*p*_end_ – *p*_start_)*V*/(*RT*), where *R* is the ideal gas constant [J mol K^–1^] and *T* is the temperature [K]. Permeance is calculated from *n*/(*A*·*t*·Δ*p*) [mol m^–2^ s^–1^ Pa^–1^], where *A* is the surface area [m^2^], *t* is the time [s], and Δ*p* is the transmembrane pressure difference [Pa]. Permeance can also be converted to permeability [cm_STP_^3^ cm cm^–2^ s^–1^ Pa^–1^] by multiplying it with the thickness of the IP film (neglecting the resistance to transport of the support). The permselectivity for a given gas pair was calculated from the ratio of their pure gas permeance values. Experiments were performed at least twice, and the reported results are the average of the obtained values.

## 3 Results and Discussion

**Figure 1:**
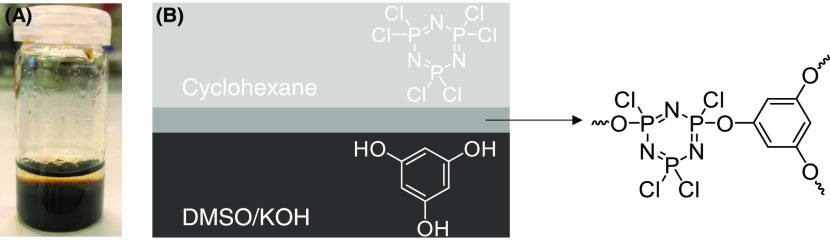
(A) Photograph of a free-standing THB-HCCP film formed at the DMSO-cyclohexane interface. (B) Illustration of the THB-HCCP network formation by the reaction of THB and HCCP.

Figures 1A and S1 are representatives of the visual observation of the localized formation of free-standing films from a reaction of THB, PDHB, or MDHB dissolved in DMSO/KOH with HCCP dissolved in cyclohexane. In all studied cases, a stable and sharp interface forms between the two solutions. As an example, Figure 1B schematically depicts the formation of the THB-HCCP film. Here, the hydroxyl groups of THB are partially deprotonated by KOH at 80 °C to form reactive phenolate anions in DMSO, causing a change in the color of the solution from light yellow to dark brown (Figure S2). During IP, phenolate anions attack the phosphor atoms of the HCCP rings, displacing the chlorine atoms in a nucleophilic aromatic substitution process. The formation and properties of the network depend strongly on the extent of conversion of the hydroxyl groups into phenolate anions that can be controlled by adjusting the molar ratio of hydroxyl groups of monomers and KOH.

**Figure 2:**
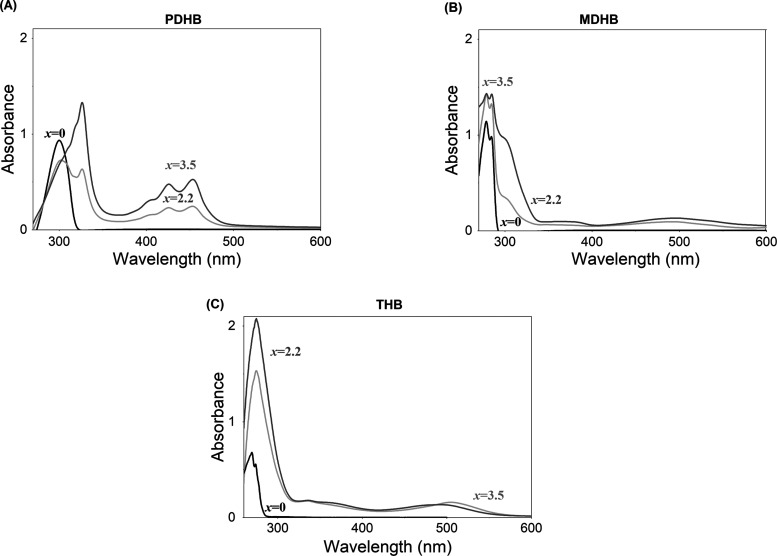
UV–vis absorption spectra of (A) 726 μM PDHB, (B) 1500 μM MDHB, and (C) 1300 μM THB in DMSO with different concentrations of KOH.

Sufficient conversion of AHCs into nucleophile phenolate anions is necessary for successful network formation. UV–vis spectroscopic measurements reveal that the extent of phenolate formation and the type of phenolate species obtained (mono, di, or trianions) depend strongly on the concentration of KOH and the structure of the employed phenols (Figure 2). In the absence of KOH (*x* = 0), the AHCs are protonated, and in the UV–vis absorption spectrum, they exhibit a characteristic sharp band below 300 nm representing an aromatic ring, which agrees with data from the literature.^29−32^ With the addition of KOH to a PDHB solution (Figure 2A), new absorptions at longer wavelengths appear (325, 400, 425, and 455 nm), which are attributed to aromatic monoanions.^33−35^ Absorptions of dianion species should appear at a wavelength of around 370 nm. This peak is not visible in the spectra due to the overlapping absorption bands that clutter the overall spectrum.^29^ Adding KOH to the MDHB solution leads to higher and broader adsorption peaks between 260 to 350 nm that are ascribed to the formation of monoanion and dianion species (Figure 2B).^31^ In addition, a new band appears at 400–600 nm. For THB (Figure 2C), the addition of KOH gives rise to new peaks around 275, 335, and 367 nm. These peaks can be attributed to the deprotonation of the hydroxyl group and the presence of dianions and trianions.^36^ For all compounds, increasing the KOH concentration (from *x* = 3.5 to *x* = 2.2) causes a substantial increase in the intensity of peaks which can be observed, implying a higher concentration of phenolate species in the solution. It is noteworthy that even at the highest concentration of KOH (*x* = 2.2), only a maximum of 45–50% of all of the hydroxyl groups are converted.

**Figure 3:**
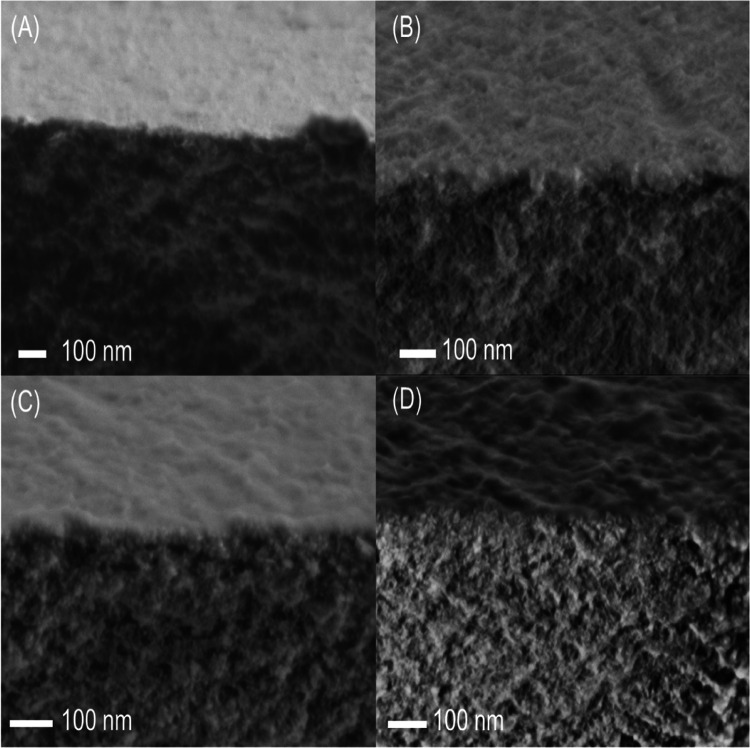
Cross-sectional FE-SEM pictures of polyphosphazene membranes. Membranes were prepared by IP of THB or MDHB in DMSO/KOH with HCCP in cyclohexane. (A) THB-HCCP, *x* = 2.2, (B) THB-HCCP, *x* = 3.5, (C) MDHB-HCCP, *x* = 2.2, and (D) MDHB-HCCP, *x* = 3.5.

Figures 3 and S3 show FE-SEM images of the cross-section and surface of TFC samples for the three different AHCs. The images reveal relatively corrugated defect-free films atop the ceramic supports, with thicknesses in the range of 20–30 nm. These thicknesses are not substantially affected by the amount of KOH in the DMSO solution and are comparable with those of IP-derived polyester nanofilms (20 nm) atop anodized alumina supports.^37^

**Figure 4:**
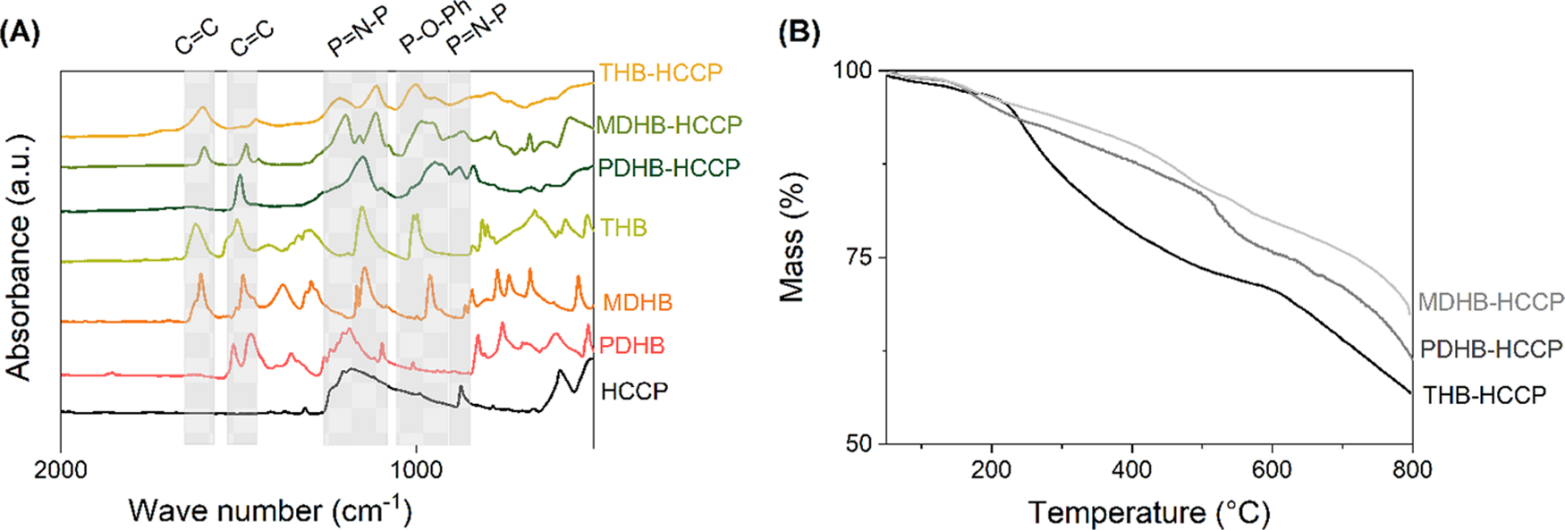
(A) FTIR spectra of free-standing polymer films formed by the IP process collected at the interface of two solvents. (B) Mass loss up to 800 °C as a function of temperature for the polyphosphazene free-standing films prepared using different hydroxyl-containing monomers.

Figure 4A depicts the FTIR spectra of the monomers and IP-derived free-standing films (*x* = 2.2). Compared to the AHC monomers, in the films, the presence of HCCP is confirmed by the additional absorption peak in the range 1100–1250 cm^–1^ due to the asymmetric P=N stretching vibration of HCCP,^23^ and the peaks around 870 cm^–1^ due to the symmetric P=N stretching vibration. The phenyl ring is apparent by the two intense peaks about 1480 and 1600 cm^–1^.^38^ The covalent connection of the AHCs and HCCP is evidenced by peaks due to the stretching vibration of Ar–O–P at 950 cm^–1^ for PDHB-HCCP and 950 and 1003 cm^–1^ for MDHB-HCCP and THB-HCCP, respectively.^39^

Figure 4B shows the mass loss of the polyphosphazene networks upon heating under N_2_. All three materials show a monotonous decrease in weight, with an onset temperature of around 200 °C. The first part of this decrease, up to ∼300 °C, is attributed to mainly the removal of adsorbed water and the residual solvent (DMSO) and some further cross-linking reactions between unreacted Cl and OH groups. This is evidenced by the release of H_2_O, CH_3_S, and Cl (Figure S4). The decrease in weight at temperatures exceeding 300 °C is distinct from the behavior of the diphenol-based materials of our previous study.^14^ For those materials, it was shown that the almost complete substitution of the Cl groups on the HCCP by Ar–O groups prevents the HCCP ring from opening at elevated temperatures, and no appreciable weight loss of these materials is observed for temperatures below 400 °C. Because the extent of substitution of Cl groups for the AHC-HCCP materials is much lower, the thermally induced opening of HCCP rings is more probable, causing the distinct thermal evolution of the mass. Hence, as a consequence of the lower degree of cross-linking and less substitution of Cl, the thermal stability of the AHC-HCCP materials is lower as compared to the diphenol-based material. By increasing the KOH concentration from *x* = 3.5 to 2.2, the extent of deprotonation of AHCs is increased, in turn affecting the polycondensation reaction kinetics. For THB-HCCP, this results in a substantially lower loss in mass with temperature (Figure S5). The difference in the residual mass at 600 °C for this material is around 10%, which is in good agreement with the number of organic bridges between HCCP cores calculated from the XRF and CN elemental analysis data, ∼8 wt %. The calculation is based on the implicit assumption that the residual mass only contains P and N, while C, O, and Cl have disappeared from the network. Using the extent of cross-linking derived from the XRF and C, N elemental analysis data, the residual masses can be calculated based on the subtraction of the molecular weights of the leaving elements from the molecular weights of the repeating unit for each *x*.

XRF data (Table 2) for free-standing AHC-HCCP films confirms the presence of the atoms of the AHCs and HCCP as well as some traces of K and S. Potassium can be present as counterions of aryloxides and/or in the form of KCl. The traces of sulfur are probably due to the incomplete removal of DMSO. The number of reacted chlorine groups, based on the ratio of Cl/P, is 1–3 out of the total of 6 Cl groups per HCCP molecule. This indicates that the AHC-HCCP networks have a low degree of cross-linking compared to our previous study.^13,14^ The fact that the materials do not readily dissolve in a variety of solvents is evidence of some degree of cross-linking. The cross-link density of the networks will be affected by the extent of deprotonation of the aryl alcohols.^18^ In our previous study, the biphenols possess well-separated OH groups that, compared to the AHCs, display lower p*K*_a_ values for the first and second deprotonation steps.^14^ This is especially the case when the bisphenols contain electron-withdrawing moieties. The more pronounced deprotonation, resulting from the lower pK_a_ values, results in a larger number of organic bridges in the network. In fact, for the diphenol-based materials, almost all Cl groups on HCCP are substituted by an aryloxide group. For the AHC-HCCP materials, the number of organic bridges is much lower and follows the order PDHB-HCCP < MDHB-HCCP ≃ THB-HCCP. This may be explained by the higher p*K*_a_ values of PDHB (p*K*_1_ = 9.9, p*K*_2_ = 11.6) compared to MDHB (p*K*_1_ = 9.2, p*K*_2_ = 10.9) and THB (p*K*_1_ = 8.0, p*K*_2_ = 9.2, p*K*_3_ = 14).^36,40^

**Table 2:** XRF Data for the Polyphosphazene Free-Standing Films

		elemental composition (%)^a^				b
sample		P	Cl	K	S	number of reacted Cl^b^
PDHB-HCCP	*x* = 2.2	32.5	48	14.5	4.6	1–2
	*x* = 3.5	34.9	53.1	9.34	2.7	1–2
MDHB-HCCP	*x* = 2.2	37.1	47.9	11.1	2.9	2
	*x* = 3.5	36.4	49.4	12	2.17	2
THB-HCCP	*x* = 2.2	36.8	48.9	8.8	4.7	2
	*x* = 3.5	37.1	41.5	13.7	7.7	2–3

a Statistical error for the data is found to be between 0.7 to 4%.

b Number of reacted Cl per HCCP is equal to the number of reacted hydroxyl-containing monomers per HCCP. This number is calculated based on the ratio of P/Cl, tacitly neglecting the presence of Cl in the form of KCl.

Since the atomic percentages of O and Cl can be influenced by the presence of KCl and the hydrolysis of P–Cl to P–OH in the presence of H_2_O, C,N elemental analysis was used to confirm the extent of cross-linking (Table S1). The number of reacted Cl per HCCP, calculated based on the ratio of C/N, is higher as compared to the XRF data but also confirms incomplete conversion of the HCCP-Cl groups.

**Figure 5:**
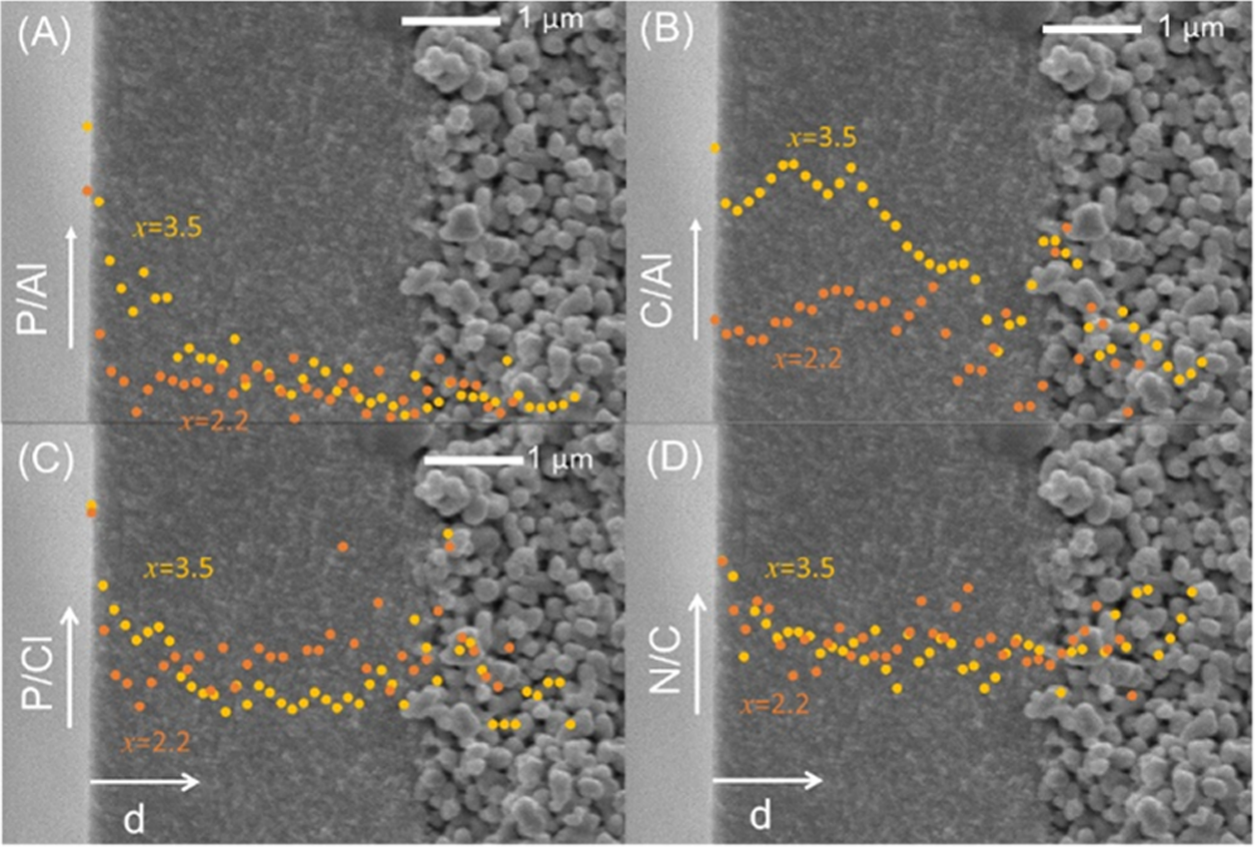
Distribution of ratios of elements over the membrane’s cross-section for THB-HCCP *x* = 2.2 and *x* = 3.5. (A) P/Al, (B) C/Al, (C) P/Cl, and (D) N/C.

Because of the intricate interplay of reaction and diffusion kinetics, in IP-prepared thin-film composites, the polymer density and the degree of cross-linking can vary with location. The EDX data in Figure 5 reveal the impact of the KOH concentration on the distribution of different elements over the cross-section of a THB-HCCP thin-film composite. The ratios P/Cl and N/C are representative of the degree of cross-linking; both these ratios are highest at the outer interface of the sample, accordant with the existence of a thin cross-linked film at this interface. The extent of cross-linking within the rest of the γ-alumina layer is similar for both KOH concentrations. The amount of polymer in the γ -alumina layer is more significant for the lowest KOH concentration. This can be rationalized by the faster reaction kinetics and hence faster film formation at high KOH concentrations, sooner resulting in impeded transport of the monomers to the reaction zone.

**Figure 6:**
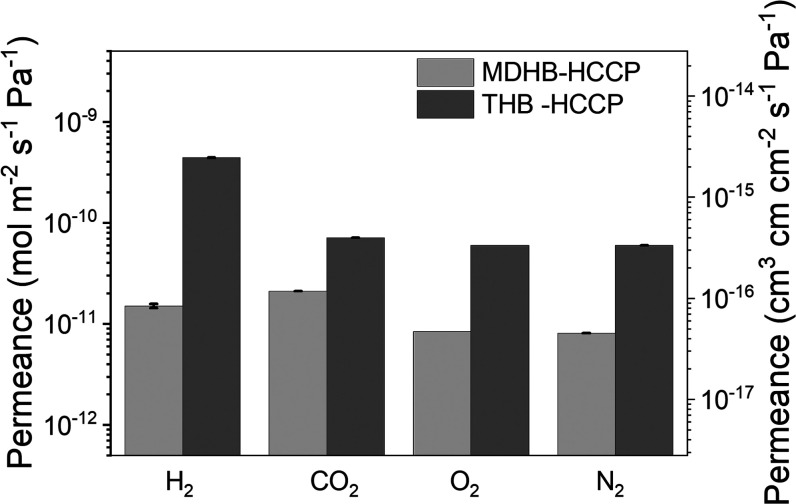
Gas permeance results of MDHB-HCCP and THB-HCCP with *x* = 2.2 at a temperature of 30 °C and transmembrane pressure of 3 bar. To aid comparison with the open literature, the data are converted into cm^3^ cm cm^–2^ s^–1^ Pa^–1^ and shown on the right axis.

The EDX data does not provide conclusive information on the effect of KOH on permselectivity or barrier properties of the thin film at the interface of the TFC. This is more evident from the gas permeance of He, H_2_, CO_2_, N_2_, and CH_4_ in the temperature range 30–200 °C for TFCs with *x* = 2.2, 3.5, and 4. For all KOH concentrations, the PDHB-HCCP films show gas permeances that are comparable to those of the bare supports (Table S2). This is attributed to a too low degree of substitution of Cl by Ar–O (Table 2), resulting in a weakly connected nonselective network. For the other two networks, the gas permeance is strongly affected by the value of *x*. For high KOH concentrations, *x* = 2.2, the THB-HCCP and MDHB-HCCP films exhibit gas barrier properties that may offer the potential for, e.g., confining the highly diffusive hydrogen gas in case of transportation and storage.^41−43^Figure 6 shows the H_2_ and O_2_ permeance of the two materials at 30 °C. The MDHB-HCCP films exhibit lower permeance as compared to THB-HCCP films. This is due to the higher molar concentration of MDHB during IP, increasing the thickness of the layer and hence the barrier properties.^44^ To aid comparison with the open literature, the data are converted into cm^3^ cm cm^–2^ s^–1^ Pa^–1^ and listed in Table 3. Both for H_2_ and O_2_, the very thin TFCs have comparable performance to existing barrier coatings or even surpass their performances.^42,45−47^ Moreover, the barrier properties of our thin film persist at elevated temperatures, as even at 200 °C, the H_2_ permeance through THB-HCCP films is 10^–9^ mol m^–2^ s^–1^ Pa^–1^ (Figure S6). For ambient temperatures, Su et al.^46^ and Tzeng et al.^45^ reported lower hydrogen permeabilities of 6.9 × 10^–19^ and 3 × 10^–19^ cm^3^ cm cm^–2^ s^–1^ Pa^–1^. These authors use completely different measurement methods and low transmembrane pressure. More importantly, they use more complex fabrication techniques involving graphene oxide and nanoclay nanosheets with high aspect ratios; the scalability of these materials is unknown. The facile IP technique used in our study is compatible with the existing large-scale industrial fabrication of reverse osmosis membranes.^48^

**Table 3:** Comparison of Different Published Barriers with Respect to Their Oxygen and Hydrogen Permeabilities

film matrix	gas	permeability	thickness	ref
poly(lactic acid)/graphene	O_2_	1.1 × 10^–14^ cm^3^ cm cm^–2^ s^–1^ Pa^–1^	210 μm	(49)
cellulose/Graphen	O_2_	0.8 × 10^–14^ cm^3^ cm cm^–2^ s^–1^ Pa^–1^	35 μm	(50)
HDPE/GO	O_2_	1.75 × 10^–14^ cm^3^ cm cm^–2^ s^–1^ Pa^–1^		(51)
cellulose nanocrystal	O_2_	1.75 × 10^–11^ cm^3^ cm cm^–2^ s^–1^ Pa^–1^	<15 μm^a^	(52)
polyelectrolyte complex	O_2_	0.013 cm^3^ m^–2^ day^–1^	1.9 μm	(53)
a high-barrier polyimide (FAPPI)	O_2_	0.43 cm^3^ m^–2^ day^–1^		(54)
PMDA-FDA polyimide	O_2_	1.01 cm^3^ m^–2^ day^–1^	75 μm	(55)
PMDA-AAPPI polyimide	O_2_	1.7 cm^3^ m^–2^ day^–1^		(56)
THB-HCCP-2.2	O_2_	3.8 × 10^–16^ cm^3^ cm cm^–2^ s^–1^ Pa^–1^	30 nm	^this work^
MDHB-HCCP-2.2	O_2_	1.1 × 10^–16^ cm^3^ cm cm^–2^ s^–1^ Pa^–1^	30 nm	^this work^
MXene-GO/poly (ethylene-*co*-acrylic acid)	H_2_	3.5 × 10^–12^ cm^3^ cm cm^–2^ s^–1^ Pa^–1^	∼10 μm	(42)
PEI/graphene oxide	H_2_	1.8 × 10^–16^ cm^3^ cm cm^–2^ s^–1^ Pa^–1^	91 nm	(57)
PDDA/graphene oxide	H_2_	3.6 × 10^–15^ cm^3^ cm cm^–2^ s^–1^ Pa^–1^	120 μm	(58)
PEN/graphite	H_2_	2.3 × 10^–14^ cm^3^ cm cm^–2^ s^–1^ Pa^–1^	70 μm	(43)
chitin	H_2_	1.3 × 10^–8^ cm^3^ cm cm^–2^ s^–1^ Pa^–1^	50 μm	(47)
PVA/silicate nanosheets	H_2_	6.9 × 10^–19^ cm^3^ cm cm^–2^ s^–1^ Pa^–1^	1.5 μm	(41)
reduced graphene oxide	H_2_	3 × 10^–19^ cm^3^ cm cm^–2^ s^–1^ Pa^–1^	30 nm	(46)
polyelectrolyte complex/clay	H_2_	4 × 10^–15^ cm^3^ cm cm^–2^ s^–1^ Pa^–1^	122 nm	(45)
THB-HCCP-2.2	H_2_	2.4 × 10^–15^ cm^3^ cm cm^–2^ s^–1^ Pa^–1^	30 nm	^this work^
MDHB-HCCP-2.2	H_2_	9.4 × 10^–17^ cm^3^ cm cm^–2^ s^–1^ Pa^–1^	30 nm	^this work^

a Because it is an anisotropic film with a flexible thickness.

**Figure 7:**
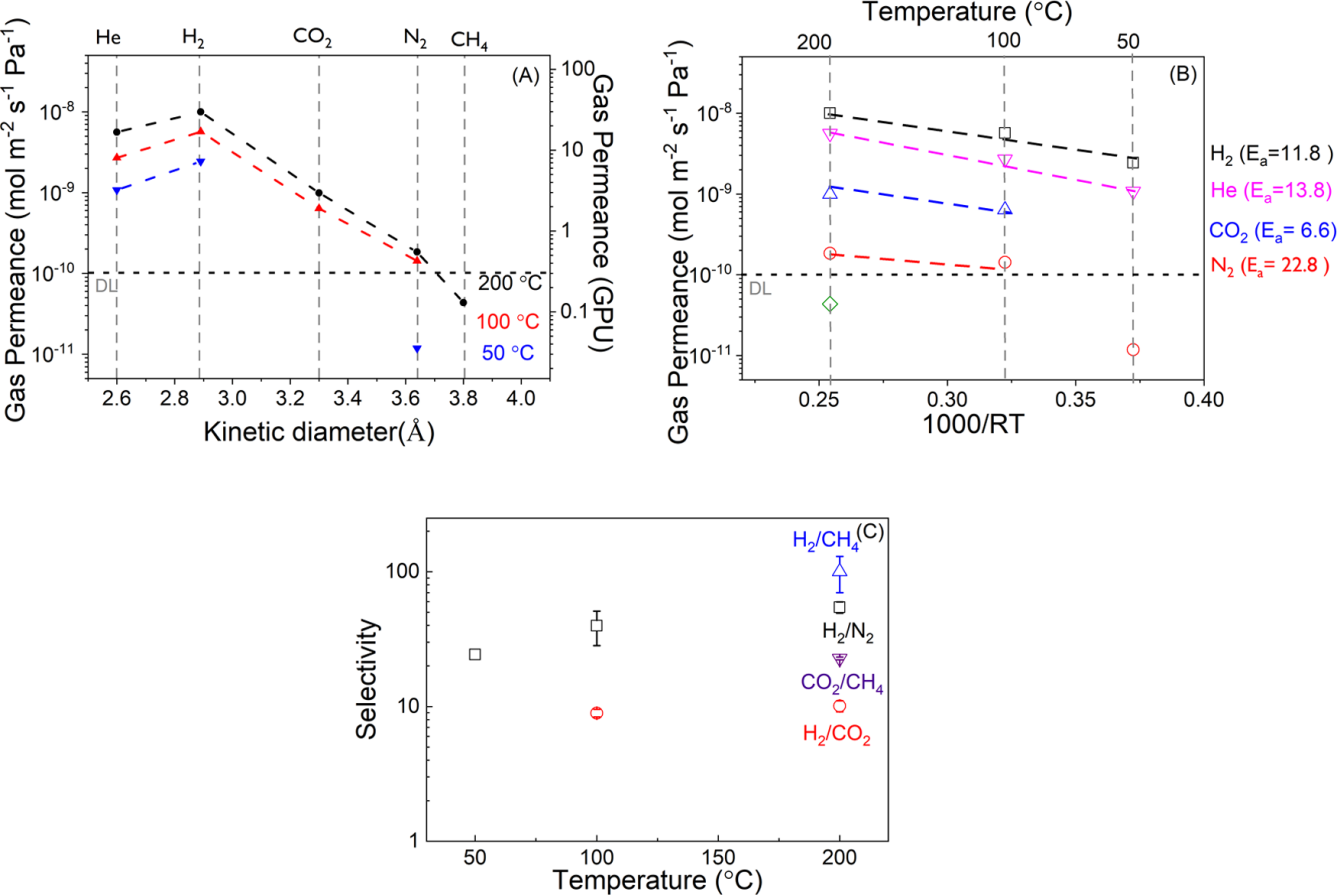
(A) Gas permeance of THB-HCCP with *x* = 4 as a function of the gas kinetic diameter for three different temperatures. (B) Arrhenius plot of pure gas permeances; the unit of activation energies in kJ mol^–1^. (C) Ideal selectivities of the membranes as a function of temperature.

A lower concentration of KOH directly affects the kinetics of the polycondensation reaction and hence affects the permselective properties of the films. In Figure 7A, single gas permeance data of THB-HCCP films, with *x* = 4, are presented as a function of gas kinetic diameter in the temperature range 50–200 °C. The increased value of *x* reduces the H_2_ barrier properties of the films. The permeance of the small gases decreases with an increasing gas kinetic diameter. This gas-sieving behavior is also observed for glassy polymers such as aromatic polyimides.^59,60^ For all gases, the permeance increases with increasing temperature, confirming that permselectivity arises from diffusion. For THB-HCCP, the activation energies obtained from the Arrhenius plots in Figure 7B follow the order N_2_ > H_2_ > He > CO_2_. This complies with the order of the kinetic diameters of the gas molecules, except for CO_2_. The distinct behavior of CO_2_ is due to its quadrupole moment and the resulting affinity for polar groups such as amines and hydroxyl groups.^61^ Increasing temperature lowers the interactions between CO_2_ and the material. The trade-off between the temperature effect on the solution and diffusion is manifested by the lower “apparent” activation energy for CO_2_.^62^ This is in line with observations for other membranes, such as thermally stable polyimide and polybenzimidazole membranes.^63−65^ The permeance of CH_4_ is below the detection limit of the setup and the activation energy of CH_4_ could not be calculated.

The membranes show high permselectivities, even at 200 °C: 10.1, 52.8, and 100 for H_2_/CO_2_, H_2_/N_2_, and H_2_/CH_4_. The H_2_/N_2_ selectivity increases with temperature, and the H_2_/CO_2_ selectivities are around 10 over the complete temperature range of 50–200 °C. Relatively few polymeric membranes have been characterized by permeation at high temperatures due to often limited membrane stability. The observed performance of THB-HCCP membranes is comparable with the performance of the poly(PMDA-POSS imide) membranes and polybenzimidazole.^65−68^ In addition, these new membranes have the following advantages: (1) They are made by interfacial polymerization, a simple and convenient technique that readily allows upscaling.^69^ (2) The THB-HCCP membranes are formed in one step; where no additional thermal treatment is needed, as is the case for polyimides.^59^ (3) Very cheap and readily accessible monomers are used for the preparation of the membranes.

## 4 Conclusions

Thin-film cyclomatrix polyphosphazene networks are prepared by nonconventional interfacial polymerization of hexachlorocyclotriphosphazene with small aromatic hydroxy compounds. As a polar phase, DMSO is used instead of water. By varying the amount of KOH in DMSO, the extent of deprotonation of the hydroxy compounds can be changed, allowing for the tailoring of the permselective properties of the thin-film composites, all the way from hydrogen/oxygen barriers to membranes with persisting hydrogen permselectivities at high temperatures. The barrier properties are obtained with high KOH concentrations, allowing for more pronounced deprotonation of the hydroxy compounds. A lower concentration of KOH results in materials with a larger free volume and faster transport of the small hydrogen molecules. These tunable materials show excellent potential for use in a broad landscape of applications, ranging from barriers for hydrogens storage and transport to high-temperature gas separation processes.

## Supporting Information Available

Free-standing film; photograph of AHC solutions; FE-SEM picture of the support; C,N elemental analysis; TGA-MS data; gas permeation of the support and PDHB-HCCP; and gas permeation performance of THB-HCCP at different KOH concentrations (PDF)
