# Supplementary material for: Nonaqueous Interfacial Polymerization-Derived Polyphosphazene Films for Sieving or Blocking Hydrogen Gas
Source: ACS Appl Polym Mater. 2023 Feb 9;5(3):1955–64. doi: 10.1021/acsapm.2c02022 (PMC10012169; doi:10.1021/acsapm.2c02022)
Supplement: Supplementary file 1 — ap2c02022_si_001.pdf [file ap2c02022_si_001.pdf]

Supporting Information

Non-aqueous interfacial polymerization derived polyphosphazene films for sieving or blocking hydrogen gas

Farzaneh Radmanesh[1], Alberto Tena[2,3], Ernst J.R. Sudhölter[1,4], Mark A. Hempenius[5], Nieck E. Benes[1]*

[1] Membrane Science and Technology Cluster, Faculty of Science and Technology, MESA+ Institute for Nanotechnology, University of Twente, P.O. Box 217, 7500 AE Enschede, the Netherlands

[2] The European Membrane Institute Twente, Faculty of Science and Technology, University of Twente, P.O. Box 217, 7500 AE Enschede, the Netherlands

[3] Surfaces and Porous Materials (SMAP), Associated Research Unit to CSIC, UVainnova Bldg, Po de Belén 11 and Institute of Sustainable Processes (ISP), Dr. Mergelina S/n, University of Valladolid, 47071, Valladolid, Spain

[4] Organic Materials & Interfaces, Department of Chemical Engineering, Faculty of Applied Sciences, Delft University of Technology, 2629 HZ Delft, the Netherlands

[5] Sustainable Polymer Chemistry, Faculty of Science and Technology, MESA+ Institute for Nanotechnology, University of Twente, P.O. Box 217, 7500, AE Enschede, the Netherlands

* Corresponding author
Email address: n.e.benes@utwente.nl (N.E. Benes).

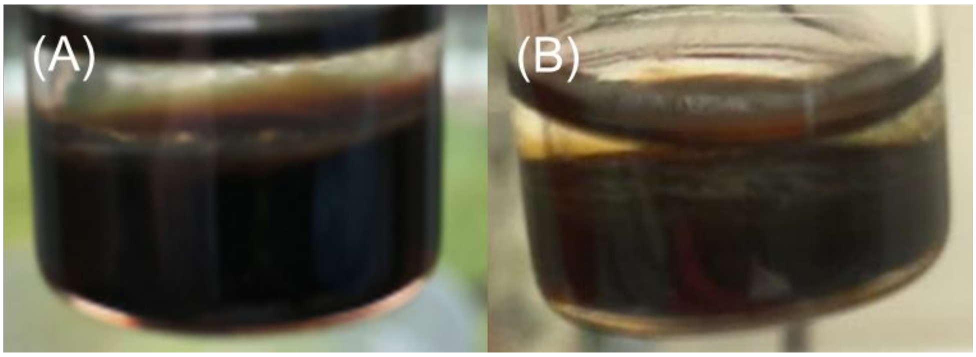

Figure S1 Photograph of free-standing (A) MDHB-HCCP (B) PDHB-HCCP films formed at the DMSO-cyclohexane interface.

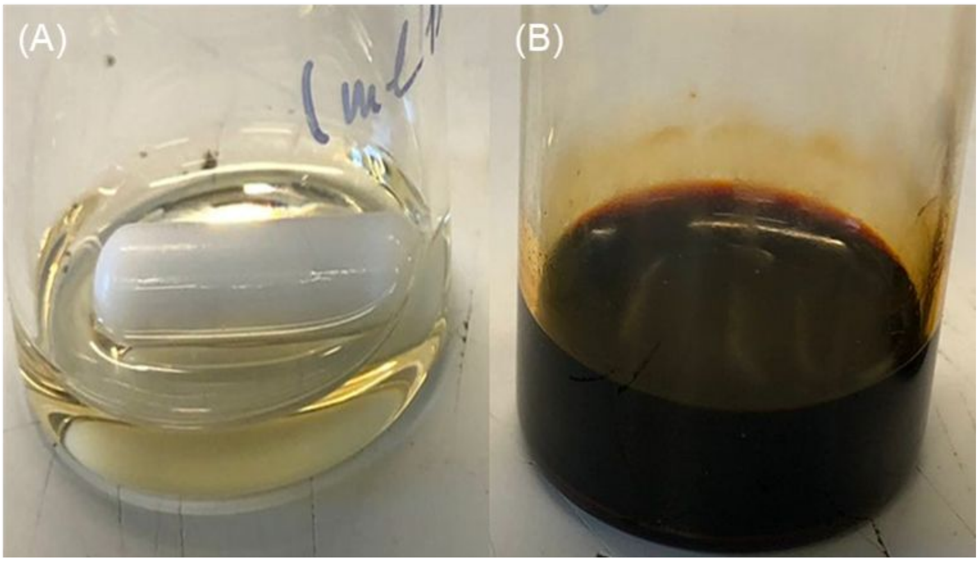

Figure S2 (A) Solution ofTHB in DMSO before addition of KOH (B) After addition of KOH and solution at 80°C for 2.5 hr.

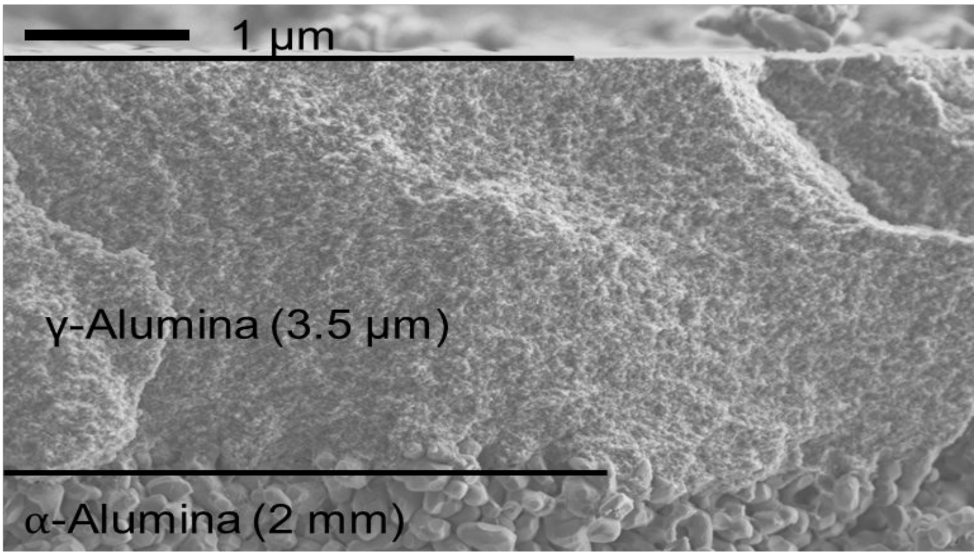

Figure S3 Cross-section scanning electron micrograph of the support used in this study. It shows the 3 .5 µm γ-Alumina layer and the 2 mm microporous α-Alumina.

Table S1 C,N elemental analysis(%) for polyphosphazene powders

Sample		Elemental concentration (%)		Number of reacted Cl[a]
		C	*N*	
**PDHB-HCCP**	*x*=2.2	30.9±0.8	8.9±0.2	3-4
	x=3.6	30.1±0.9	8.7±0.2	3-4
**MDHB-HCCP**	*x*=2.2	32.7±0.2	8.2±0.3	4
	x=3.6	32.5±0.2	8.1±0.3	4
**THB-HCCP**	*x*=2.2	19.9±0.5	8.8±0.2	2-3 (2.3±0.1)
	x=3.6	22±0.4	8.4±0.1	2-3(2.6±0.1)

[a]The number of reacted Cl groups is equal to the number of reacted hydroxyl contained monomers per HCCP. This number is calculated based on the ratio of C/N.

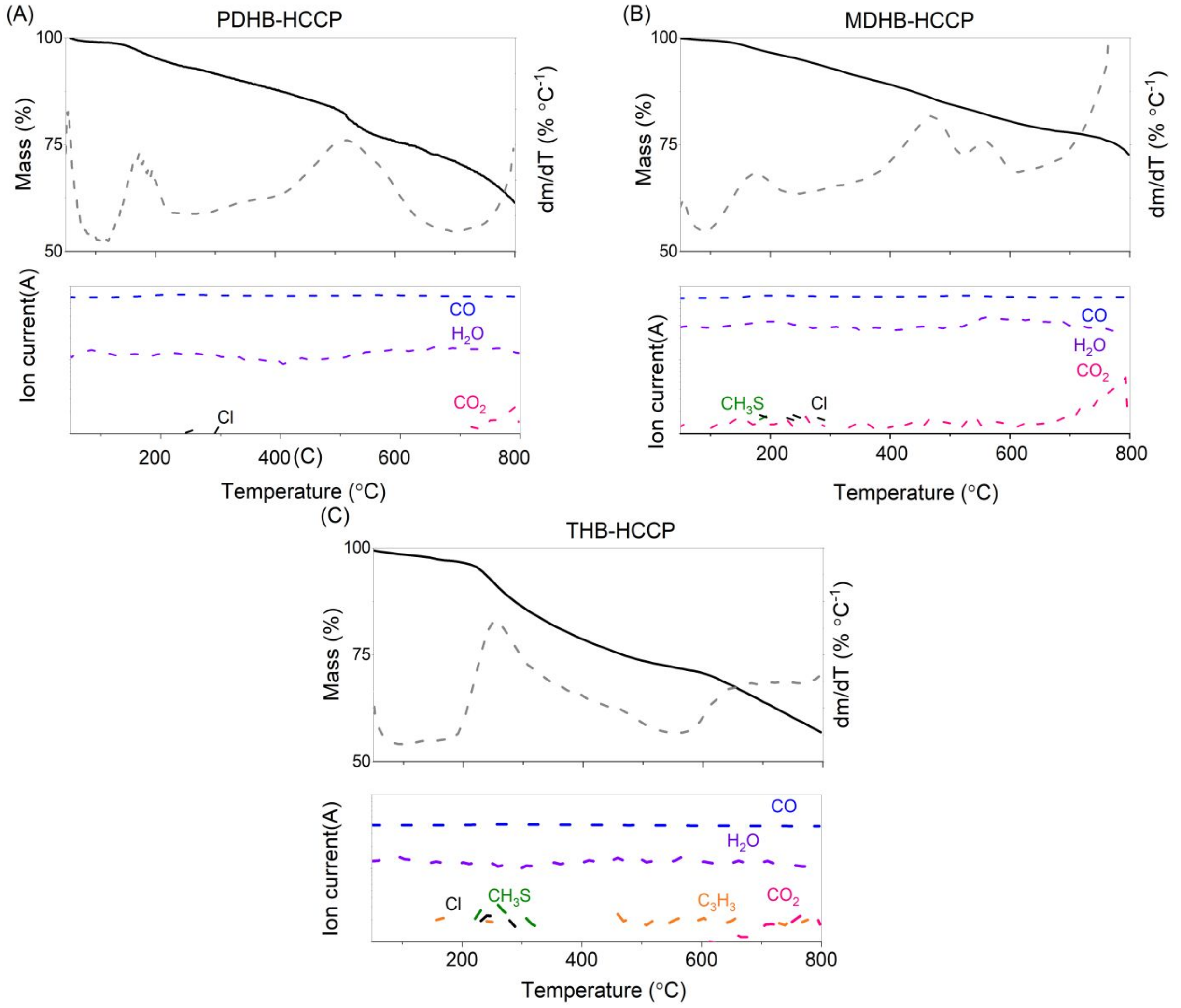

Figure S4 The mass loss (top panels) and the evolved gases (bottom panels) as a function of temperature for the polyphosphazene free-standing films prepared using different hydroxyl-containing monomers, obtained with a heating rate of 10 °C min^{-1}. (A) PDHB-HCCP, (B) MDHB -HCCP, and (C) THB-HCCP.

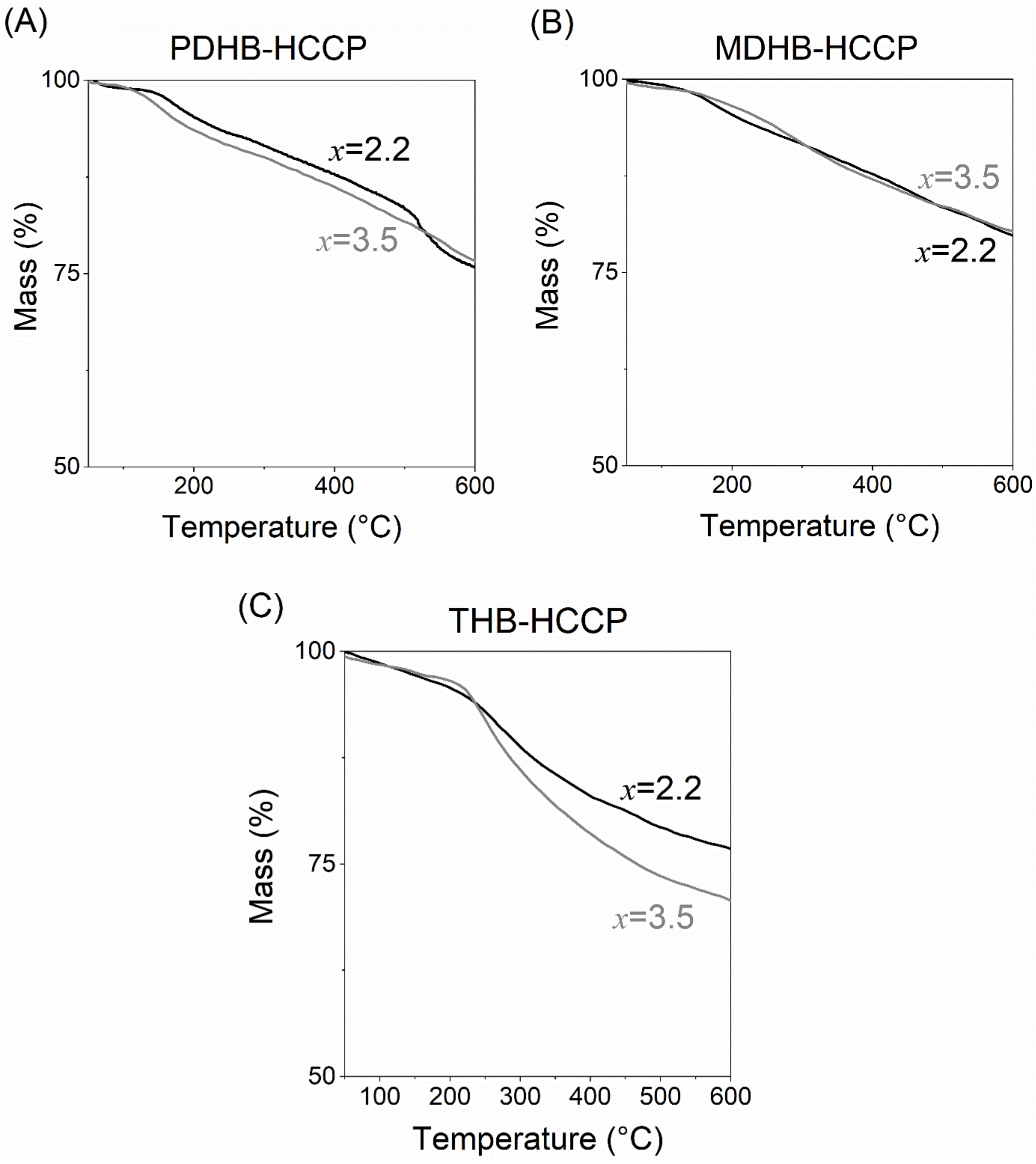

Figure S5 Comparison of the changes in sample mass of prepared free-standing films as a function of the temperature for the different KOH concentrations, x, A) PDHB-HCCP, (B) MDHB -HCCP, and (C) THB-HCCP. All samples were heated under N2 at 10 °C min^{-1}.

Table S2. Comparing the single gas separation properties of the supports and PDHB-HCCP membrane at 2 bar and 30 °C.

Gases	Gas permeance (mol m^{-2} s^{-1} Pa^{-1})	
	**Support**	**PDHB-HCCP**
**He**	$1.1□10^{-6}$	$1.1□10^{-6}±4.2□10^{-9}$
**H_2**	$2□10^{-6}$	$2□10^{-6}±1.3□10^{-8}$
**CO_2**	$6.1□10^{-7}$	$6.2□10^{-7}±1.9□10^{-8}$
**N_2**	$5.7□10^{-7}$	$5.8□10^{-7}±5□10^{-9}$
**CH_4**	$9.7□10^{-7}$	$9.8□10^{-7}±1.5□10^{-9}$

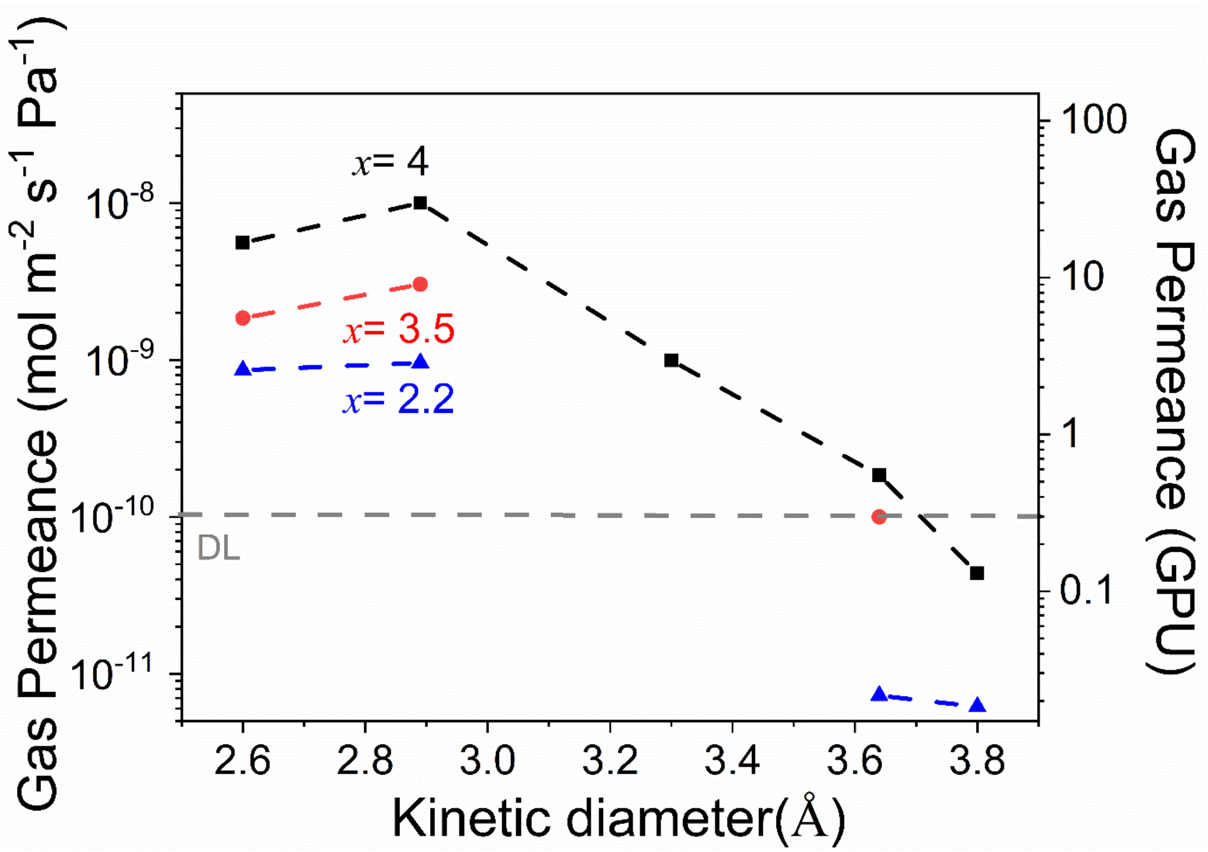

Figure S6 Gas permeance of THB-HCCP as a function of gas kinetic diameter at 200 ˚C for three different x.
